# Identifying corridors of river recovery in coastal NSW Australia, for use in river management decision support and prioritisation systems

**DOI:** 10.1371/journal.pone.0270285

**Published:** 2022-06-23

**Authors:** Danelle Agnew, Kirstie Fryirs

**Affiliations:** School of Natural Sciences, Macquarie University, North Ryde, NSW, Australia; University of Bucharest, ROMANIA

## Abstract

By connecting corridors of river recovery, resilience can be built into river systems to mitigate against future floods and droughts driven by anthropogenic disturbance or climate extremes. However, identifying where these corridors can be built is still lacking in river management practice. The Open Access NSW River Styles database contains comprehensive information on geomorphic river condition and recovery potential. The database can be used to systematically analyse where corridors of river recovery could be created via conservation or rehabilitation. Analysis was undertaken in ArcGIS using the recovery potential layer along 84,342 km of freshwater stream length, across 20 catchments of coastal NSW. We identified 4,905 km of reach connections, defined as an upstream to downstream section of river that is connected end-to-end, and 17,429 km of loci connections defined as more isolated sections of river from which recovery can be seeded and extended into adjacent reaches. There was significant spatial variability in the types and lengths of connections made across the catchments. Some catchments have significant potential to build corridors of recovery along large sections of river, whereas other catchments are more fragmented. These results provide practitioners with a user-friendly distillation of where river conservation and rehabilitation activities could be focussed when working with river recovery in practice. Combined with local on-ground knowledge, this information forms an important input to evidence-based prioritisation and decision making in river management.

## Introduction

Rivers are the natural corridors and arteries of the landscape, and fluvial corridors are integral connectors within large-scale landscape and ecosystem corridors that provide multiple ecosystem benefits and services [1]. However, centuries of climate and anthropogenic disturbance have already caused drastic changes to riverine structure, function and health, globally. In the United Nations Decade of Ecosystem Restoration [2], it is now critical that river management use assisted recovery to improve the resilient capacity of rivers so that the ecosystems and the societies that depend on them can sustainably adapt going forward [3–6]. One way that this can be achieved is by building corridors of river recovery across the landscape. River recovery is defined as the trajectory of change a reach takes towards an improved condition [7]. In geomorphic terms this includes improvement in both the physical structure and function of a river [7].

Globally, nature-based approaches and solutions (NBS), which seek to mimic or use natural processes to preserve, rehabilitate or create a range of ecosystems, have been integrated into regulatory policy and decision-making across a broad range of environmental concerns [8] and organisations [9, 10]. Concurrently, in many parts of the world, river management philosophy has shifted from an engineering based approach to an ecosystem based approach to rehabilitation and restoration [11, 12] with preferred rehabilitation strategies being process-based, self-healing, and recovery-based [13–20]. Working with recovery in this ‘era of NBS’ shifts the emphasis from treating the most degraded parts of river systems to concentrating rehabilitation efforts where river recovery is already occurring or can be enhanced and assisted to trigger positive feedbacks beyond the reach scale [13, 15]. However, for this to be achieved requires that river managers are working at-scale (i.e. catchment or regional scales) and considering the fluvial corridor as a fundamental management unit (i.e. moving away from independently managed reaches to sequences of reaches at large scales) [7, 21, 22].

An integral part of a landscape is its networks or linkages of corridors that occur across ecosystems [23]. Operating at different scales, corridors are areas within the landscape which provide the capacity for exchange, dispersal and migration passageways for matter and organisms [24]. Variously known as ecological or dispersal corridors, wildlife movement corridors, or landscape linkages [23, 25], corridors can be environmental (characterised by local vegetation, geology or fluvial conditions), remnant (a response to disturbance) or introduced (created by humans) [26]. One common characteristic is that corridors sustain critical biodiversity, habitat and ecological function [27, 28]. Processes that link landscape units, or corridors, adjust and operate at various spatial and temporal scales, allowing species to move freely within their natural range and complete their lifecycles [29, 30]. Natural corridors prevent ecosystems and populations from becoming isolated or extinct by maintaining functional connectivity, and aiding to mitigate the adverse impacts of habitat fragmentation [25]. Corridors also allow populations to shift distribution in response to natural disturbance events, human land use and climate change [23, 27, 31, 32]. Corridors can have a range of physical attributes, narrow or wide, straight or curved, and short or long, can be connected temporally or spatially with varying degrees of connection or disconnection to adjacent corridors [26]. Interconnected corridors can form networks or ranges in the landscape [27]. Climate change-focused conservation policies recognise the need for expansion of corridor networks as a central adaptation strategy for environmental condition, health and biodiversity protection [27, 30]. Indeed, the International Union for Conservation of Nature (IUCN) published comprehensive guidelines on ecological conservation that recommend the formal recognition of ecological corridors to enhance conservation networks and promote more coherent efforts to ensure their effective protection at sub-national to international scales [1].

River systems, which are natural landscape arteries, form significant riparian and fluvial corridors [33]. Fluvial corridors provide a wide range of ecosystem services, including the *provisioning* of potable water, food and renewable energy, *regulation* of water and soil quality, habitat and biodiversity, and *cultural* benefits for community and recreation [22, 34–37]. Within fluvial corridors dynamic interactions between water, sediment, vegetation, fauna and matter in floodplain, riparian and active channel zones create a dynamic physical (geomorphic) habitat template with the required elements to sustain aquatic flora and fauna structure and function [12, 38–44]. These synergistic interactions between processes, disturbances and form can be used to characterise and identify fluvial corridors [29, 45–47].

At any position a fluvial corridor can become broken, disjointed or fragmented, where ‘poor condition’ reaches of river are found between ‘good condition’ reaches of rivers. This fragmentation can occur as a result of natural and/or anthropogenic disturbances [33]. Identifying where corridors remain ‘intact’ and where fragmentation has occurred provides an information base with which to determine how to best protect them, or build them [30, 45, 48]. Examining corridor and fragmentation characteristics of river systems also provides insights into the factors which facilitate or constrain recovery, which is critical for effective river management [33, 49].

With extensive catchment systems and resource allocation limits, one challenge practitioners and decision-makers face is how to systematically identify the most suitable river reaches to work at, so they can be prioritised and targeted for rehabilitation. A range of feasibility and environmental criteria and methods can be used to analyse and identify reaches that connect to each other to create river corridors for conservation and rehabilitation. This analysis can be based on current condition, or the potential for adjustment and/or recovery of riparian, geomorphic or hydrologic characteristics [30, 45, 48, 50–53]. Protecting reaches that are currently in good condition and rehabilitating those that will enhance the recovery potential of the corridor, generally yields more effective rehabilitation outcomes than expending effort and resources on rehabilitating more degraded streams that are often the cause of corridor fragmentation [53].

**Fig 1:**
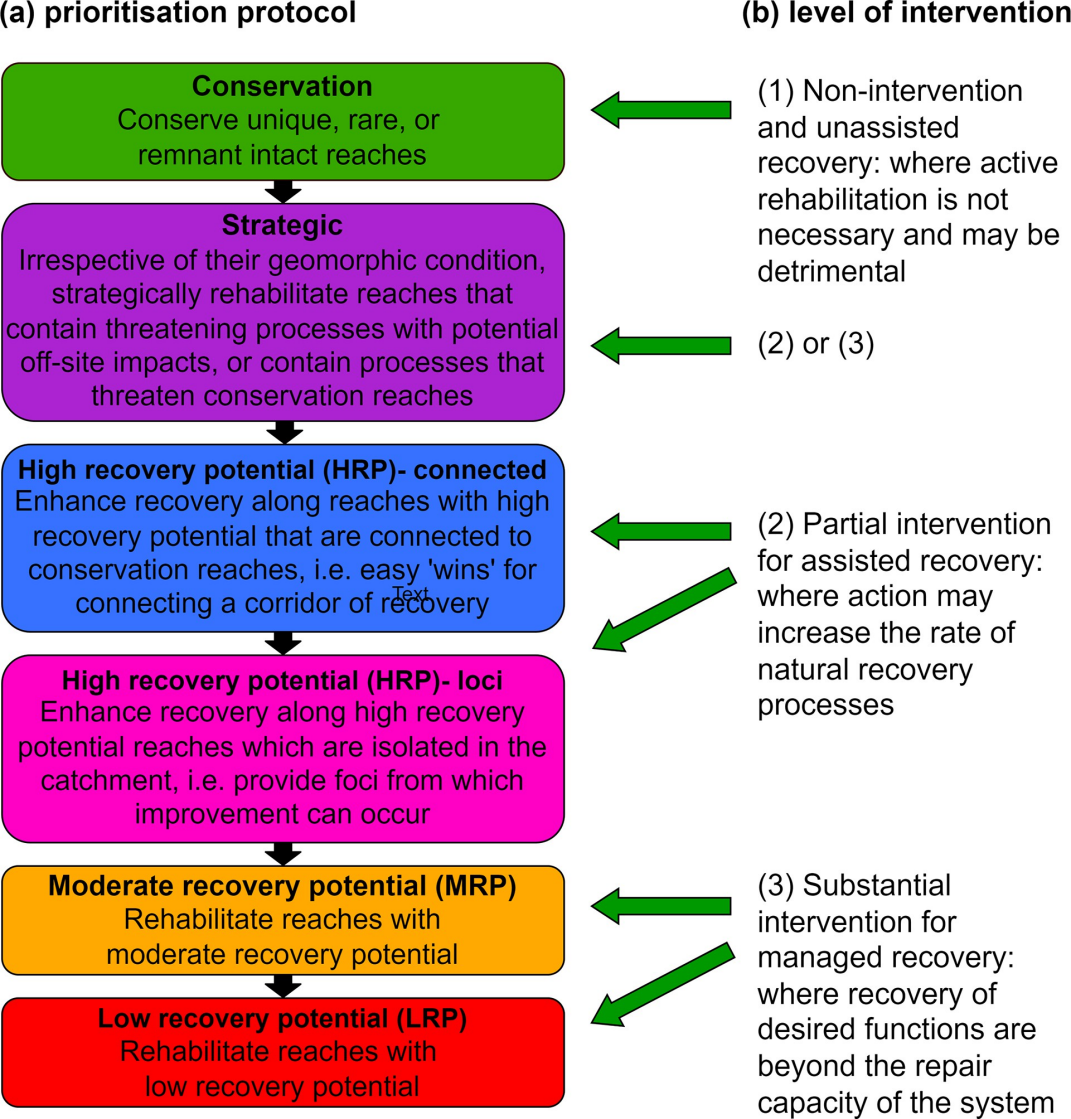
Prioritisation protocol of river reaches and level of intervention required. (a) Prioritisation protocol based on recovery potential and (b) Level of intervention required to enhance recovery and improve condition. Adapted from the River Styles Framework, Stage 4 [57].

If large, publicly available datasets of river diversity, condition and recovery potential are available, various algorithms can be run in Geographical Information Systems (GIS) to undertake corridor analysis [21, 54]. Such analysis is a first step that uses available, open source data to identify a pool of candidate reaches for intervention (from a much larger dataset), upon which more detailed and case-specific criteria can then be applied. In New South Wales (NSW), Australia, there is an opportunity to perform such analyses using the state-wide NSW River Styles database; hereafter the database [55]. The database contains comprehensive geomorphic information for over 216,000 km of stream length [19]. NSW coastal catchment sizes range from 520 km^2^ (Brunswick) to 22,716 km^2^ (Clarence). Streams from headwaters to the tidal limit have been included in the analysis. In this study, we use the geomorphic recovery potential layer in the database to undertake geomorphic corridor analysis in all coastal catchments of NSW (see [19]). In the River Styles Framework, geomorphic recovery potential is defined as the likelihood that a river reach will improve its condition over a management timeframe of 50–100 years [7, 14, 56]. Classes of recovery potential occur along a gradient from Conservation to Low Recovery Potential (LRP), as outlined in Fig 1 [57].

At the top of the recovery potential gradient are Conservation reaches which are intact and may be unique or rare. These reaches do not require active rehabilitation intervention, relying only on unassisted recovery (Fig 1B). This is followed by Strategic reaches that contain threatening geomorphic processes (e.g. headcuts or sediment slugs) that may have off-site consequences for adjacent Conservation or High Recovery Potential (HRP) reaches [57]. Therefore, these reaches require rehabilitation intervention if the integrity of the system is to be maintained. Strategic reaches may require either partial or substantial intervention depending on the type and severity of threatening process that is occurring and the condition of the reach. Next are HRP reaches, which are generally in good geomorphic condition and may already be connected to other reaches in good condition, thereby presenting excellent opportunities to augment and enhance recovery along a corridor. Alternatively, HRP reaches may be isolated (called loci) from which recovery can be seeded and extended into adjacent reaches [7]. When working with recovery in practice, most interest is often placed on reaches characterised as Strategic, HRP connected and HRP loci because these reaches tend to require minimal intervention (e.g. weed management) or no intervention at all (called the opt-out and leave it alone and monitor it approach by Fryirs, Brierley [14]). This presents opportunities for sustainable environmental improvement at lower investment cost. Continuing along the gradient, Moderate Recovery Potential (MRP) and Low Recovery Potential (LRP) reaches have limited potential to improve their geomorphic condition over a 50–100 year timeframe unless substantial intervention occurs. In these cases, it is sometimes appropriate to leave these reaches alone until they start to show signs of recovery, before intervening to enhance that recovery.

When rolled out across catchments, regions, or States, the distribution of river reaches of different recovery potential can be mapped along streamlines, and pattern analysis undertaken to determine where corridors of river recovery occur or can be built. In this work we define a reach connection as an upstream to downstream section of a river that is connected end-to-end, within a single stream, forming an extended single section of river. For example, working from upstream to downstream, upstream reaches can be rehabilitated to create positive off-site impacts and connection to downstream Conservation or HRP reaches [14, 58] (Fig 1A). Alternatively, rehabilitation can be undertaken by working out from loci connections within the catchment. Loci connections are upstream to downstream sections of a river that are connected both end-to-end within an individual stream, and also from surrounding tributaries which join this stream [57]. For example, working out from loci could involve treating stream incision in a Strategic reach, or working outwards from HRP reaches into more degraded reaches [14, 59] (Fig 1A). By working to extend these corridors, use of the HRP reaches is maximised [57]. The major difference between reach and loci connections is that loci also include tributary junctions along the main stem. In this paper we operationalise this protocol to undertake pattern analysis to identify reach and loci connections and map where corridors of river recovery occur, or could be rebuilt, in all coastal catchments of NSW. This study has five aims:

1. Use an existing State-wide database to identify the location of, and quantify the extent of, river reaches with high and strategic potential for geomorphic recovery in coastal river catchments of NSW.
2. Systematically determine the geomorphic recovery potential of adjacent (upstream and downstream) reaches to HRP and Strategic reaches.
3. Identify reaches that can be connected to build corridors of high recovery potential.
4. Identify reaches that form loci from which recovery potential can be built.
5. Discuss implications and use of the findings for prioritisation and decision support systems in river management practice.

## Methods

The full GIS workflow for undertaking corridor analysis using a large-scale database provides the step-by-step process developed and used. We intend to provide this workflow in the future so others can apply similar analyses in their work. Here we only provide a summary of the method and workflow.

Corridor analysis has been undertaken by running various algorithms to identify patterns and sequences in the database [21, 54]. Using ArcMap, the recovery potential layer was systematically analysed to identify various reach and loci connections. For coastal catchments of NSW, the database contains freshwater and tidal reaches. 119,392 freshwater reaches, ranging from <1 m to ~105 km in length, and averaging 706 m, were analysed. Tidal reaches were excluded from the analysis. To reduce processing time, reaches in the same recovery potential class that occur end-to-end along a single stream were merged, creating 41,870 reaches ranging from <1 m to ~155 km, and averaging 2 km. For loci connections, a threshold reach length of 1000 m (1 km) was used to reduce the number of connections made. A ‘target’ reach was selected according to its suitability for rehabilitation, for example, a Strategic reach, and then various reach and loci connections were identified according to their proximity to this ‘target’ reach. In this analysis we have run 13 recovery potential combinations we believe to be of most interest to river managers in NSW (Table 1) out of a total of 80 possible permutations that could be produced with this particular database. However, the workflow can be run with any combination that may be of interest to a user. The user needs to ‘tell’ the workflow what combinations and sequences to look for.

**Table 1:** Reach and loci connections identified from the NSW River Styles recovery potential layer using ArcMap.

Target recovery potential	Connection type	Selection criteria
***Reach connections***		
High Recovery Potential (HRP)	HRP between Conservation	Rehabilitation of HRP reach will enhance recovery along corridor.
Strategic	Strategic upstream of HRP	Rehabilitation of Strategic reach, for example sediment slug, to create positive connections to downstream HRP and/or Conservation reach.
	Strategic upstream of Conservation	
	Strategic downstream of Conservation	Rehabilitation of Strategic reach, for example headcut, to protect upstream Conservation and/or HRP reach from negative impacts.
	Strategic downstream of HRP	
	Strategic between HRP	Rehabilitation of the Strategic reach will connect up a corridor with enhanced recovery potential and create positive off-site impacts.
	Strategic between Conservation	
Low Recovery Potential (LRP)	LRP upstream of HRP	LRP reach likely to have negative impacts on downstream HRP and/or Conservation reach. Rehabilitation may trigger recovery in the LRP reach to protect downstream reach.
	LRP upstream of Conservation	
***Loci connections***		
Strategic	Strategic surrounded by LRP and/or Moderate Recovery Potential (MRP)	Working outwards to trigger geomorphic recovery in adjacent LRP and/or MRP reaches, and protect the Strategic, HRP and Conservation reaches.
High Recovery Potential (HRP)	HRP surrounded by LRP and/or MRP	
Conservation	Conservation surrounded by LRP and/or MRP	
Strategic	Strategic surrounded by HRP and/or Conservation	Rehabilitating of Strategic reach to protect surrounding HRP and/or Conservation reaches.

To identify reach connections end-to-end and upstream-downstream, along single channel streams, a publicly available (Geosciences Australia) 30 m spatial resolution, 1 second Shuttle Radar Topography Mission (SRTM) Digital Elevation Model (DEM-H) was used [60]. The DEM was used to determine change in elevation and thus flow direction along the river course. Other DEMs with coarser resolutions were trialled but DEM_H yielded more accurate results. The more complex loci connections required identification of both end-to-end, upstream-downstream, and adjacent tributary connections.

**Fig 2:**
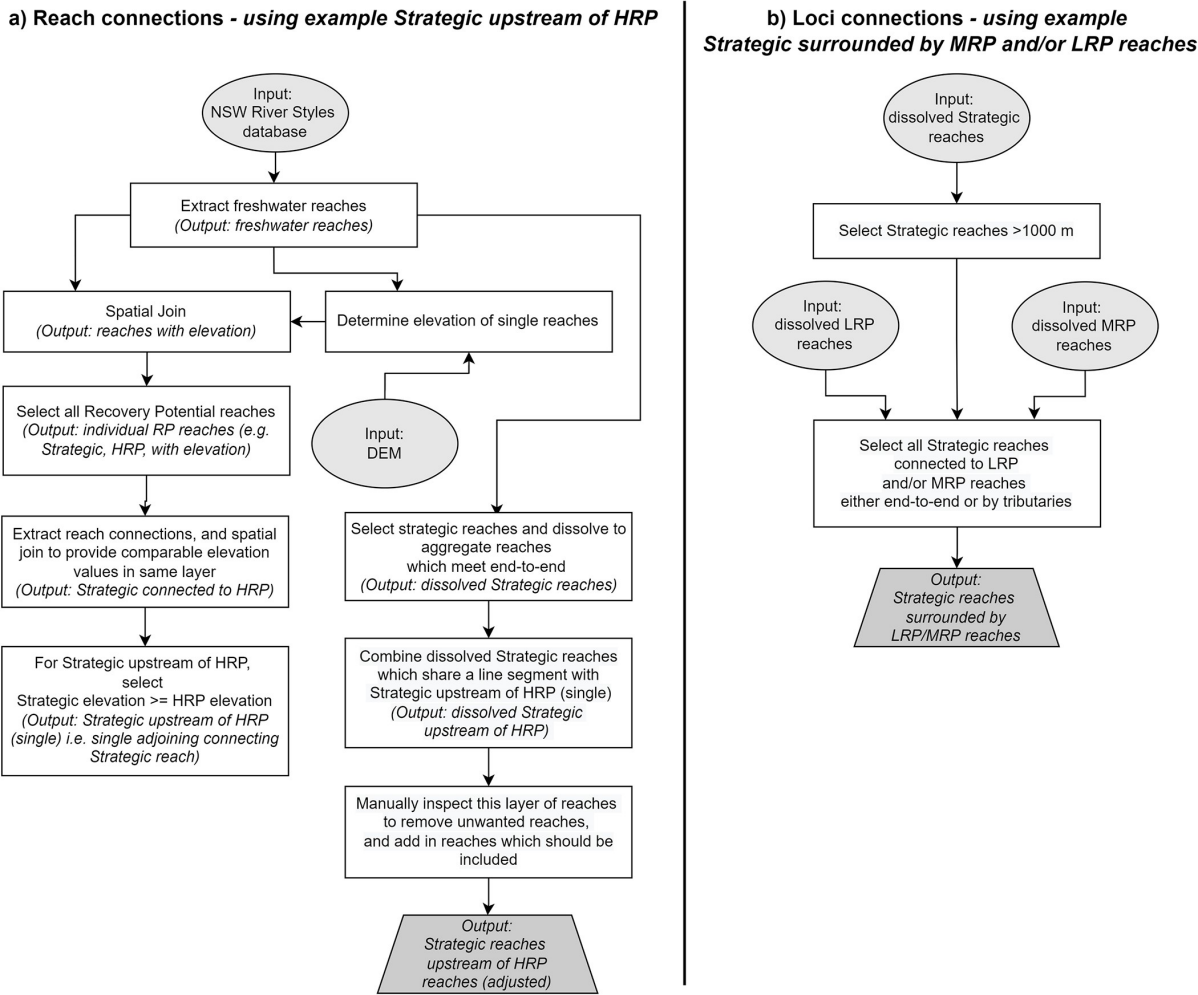
Summary workflow for extracting reach and loci connections. Workflow shows a) Strategic reaches upstream of High Recovery Potential reaches and b) Strategic reaches surrounded by LRP and/or MRP reaches. Different recovery potential combinations can be used, depending on the aims of the analysis being conducted by a user.

For reach connections, the workflow summarised here uses the identification of Strategic reaches which are upstream of High Recovery Potential reaches as the target (Table 1, Fig 2). For loci connections, we target Strategic reaches surrounded by LRP and/or MRP reaches (Fig 2).

## Results

**Fig 3:**
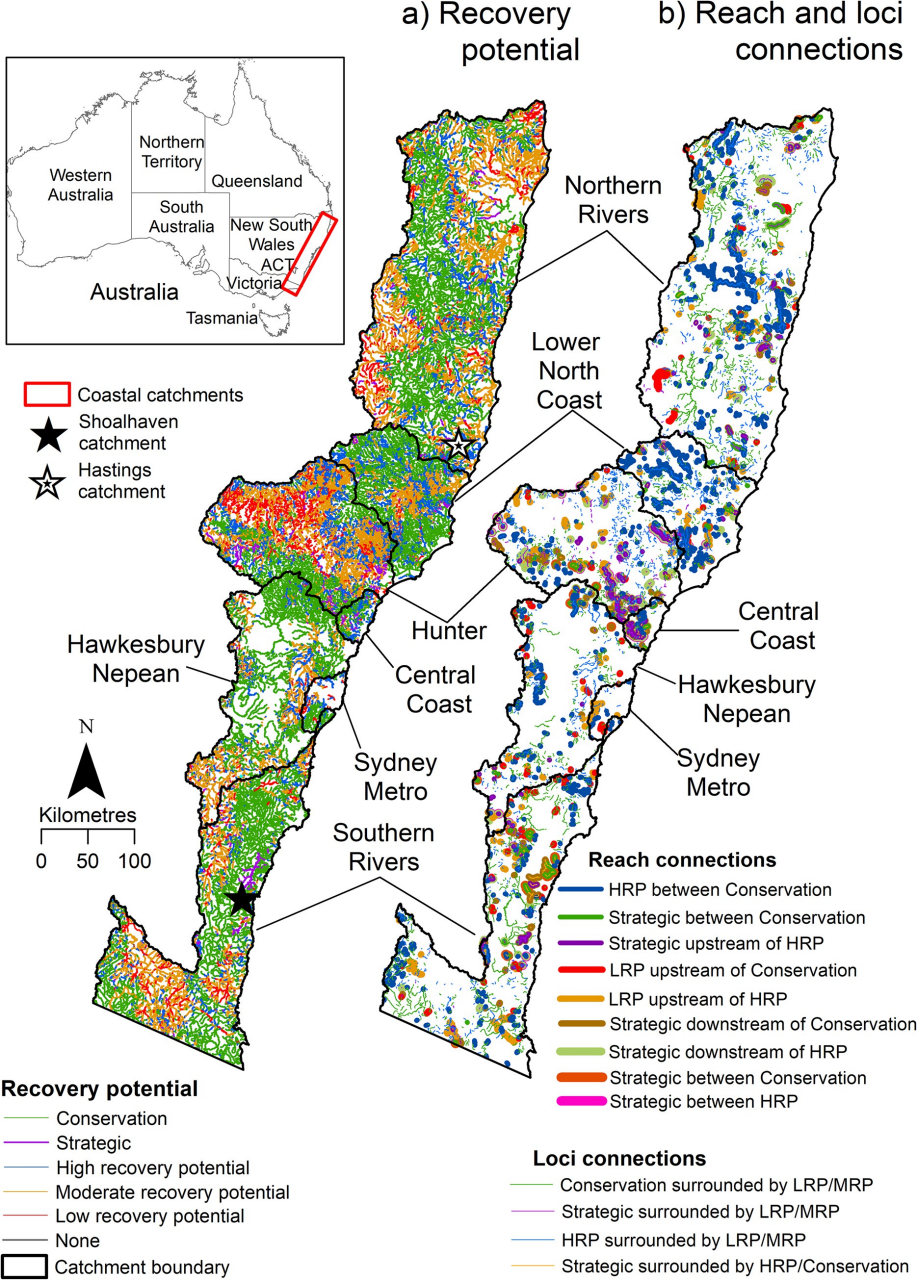
NSW coastal catchments showing recovery potential, and reach and loci connections. (a) Recovery potential derived from the NSW River Styles database. (b) Reach and loci connections identified in this study. Hastings, Hunter and Lower North Coast (H-LNC) and Shoalhaven catchments highlighted.

NSW coastal catchments extend longitudinally over 1050 km from the Queensland to Victorian border and inland up to 200 km to the Great Dividing Range. They contain 84,343 km of freshwater stream length, in an area of 129,222 km^2^ (Fig 3). Eighty-three percent of the rivers are Confined or Partly confined [19]. The catchments of the Hastings in the Northern Rivers region, Hunter and Lower North Coast (H-LNC) on the Mid North coast and Shoalhaven in the Southern Rivers region encompass the full range of selected reach and loci combinations, and have been chosen for display purposes to demonstrate the output from this analysis (Fig 3).

In Fig 3A, the recovery potential layer contains a significant volume of stream length for NSW, with dense and complex recovery potential patterns. However, Fig 3B shows how the corridor analysis has successfully identified the reach and loci connections, providing a more user-friendly distillation and focus that can now be used for prioritisation and decision-support.

Across all coastal catchments of NSW, Strategic, HRP and LRP reaches comprise 1,756 km, 13,185 km and 5,832 km of stream length, respectively (Fig 3 and Table 2). Reach connections ranged in length from <1 km to 42 km, averaging 2.6 km. Loci connections ranged in length from <1 km to 155 km, averaging 2.6 km (Table 2). S1 Table provides recovery potential data, and reach and loci connections for each coastal region for NSW.

**Table 2:** Recovery potential, and reach and loci connections for all NSW coastal catchments and catchments selected for display in this paper.

Reach and loci connections	All NSW coastal catchments			H_LNC	Hastings	Shoalhaven
*a) Reaches by Recovery Potential*	total stream length km^a^	mean reach length km^a^	# of dissolved reaches^a^	total stream length km		
Conservation	32,567.6	2.7	12,174	7,315.2	959.4	301.6
Strategic	1,755.9	1.8	984	894.3	67.8	61.9
HRP	13,185.4	1.8	7,204	5,454.4	460.5	425.4
MRP	22,117.0	2.1	10,729	7,676.7	444.7	878.5
LRP	5,832.0	1.9	3,098	2785.9	1,674.1	2,641.2
Null^b^	8,884.8	1.2	7,680	421.9	17.5	209.2
***Totals***	***84*,*342*.*7***	***2*.*0***	***41*,*869***	***24*,*548*.*3***	***3*,*624*.*1***	***4*,*517*.*7***
***b) Reach connections***						
HRP between Conservation	2,418.4	3.1	792	633.6	56.9	137.2
Strategic upstream of HRP	506.4	2.1	243	224.4	16.8	3.8
Strategic upstream of Conservation	246.0	2.8	87	36.0	44.7	59.7
LRP upstream of HRP	427.5	1.7	248	135.6	1.4	18.9
LRP upstream of Conservation	241.3	2.2	110	11.6	1.9	48.7
Strategic downstream of Conservation	556.1	3.1	179	146.3	8.5	32.8
Strategic downstream of HRP	508.9	2.5	202	310.4	19.0	17.7
***Total reach connections***	***4904*.*6***	***2*.*6***	***1861***	***1497*.*9***	***149*.*2***	***318*.*8***
***Other reach connection types (stream length already included in above categories)***						
Strategic between HRP	205.6	2.6	78	129.9	0.0	1.6
Strategic between Conservation	72.9	1.7	42	13.5	0.0	13.9
Strategic reach connections (km and % of total Strategic reaches)	1,391.5	79.3% of total		597.2	88.9	129.5
HRP reach connections (km and % of total HRP reaches)	2,418.4	18.3% of total		640.5	56.9	137.2
LRP reach connections (km and % of total LRP reaches)	668.8	11.5% of total		121.2	3.2	67.7
***c) Loci connections*** ^c^						
Strategic surrounded by LRP/MRP	935.2	4.1	227	468.9	36.0	39.3
HRP surrounded by LRPMRP	7,071.4	3.8	1,859	2,391.7	284.7	227.1
Conservation surrounded by LRP/MRP	8,476.5	4.4	1,942	1,034.8	438.2	809.2
Strategic surrounded by HRP/Conservation	945.6	4.1	229	308.2	55.8	43.5
***Total loci connections***	***17*,*428*.*6***	***4*.*1***	***4*,*257***	***4*,*203*.*5***	***814*.*7***	***1*,*119*.*1***
Strategic loci connections (km and % of total Strategic reaches)	1,394.9	79.4% of total		596.0	58.0	57.2
HRP loci connections (km and % of total HRP reaches)	7,071.4	53.6% of total		2,391.7	284.7	227.1
Conservation loci connections (km and % of total Conservation reaches)	8,476.5	26.0% of total		1,034.8	438.2	809.2

^a^Freshwater stream length is shown for (a) reaches by recovery potential, (b) reach connections, and (c) loci connections for all NSW coastal catchments and the Hastings, Hunter and Lower North Coast (H-LNC) and Shoalhaven catchments. Mean reach length (km) and number of dissolved reaches is shown for all NSW coastal catchments.

^b^Null reaches are streams where recovery potential has not been assessed. These include streams inside National Parks/Reserves, dams, weirs, lakes, urban streams, engineered channels and diversions, or where assessment requires further field verification.

^c^For loci connections, only Conservation, Strategic and HRP reaches >1000 m were included in the analysis, to reduce the number of short reaches that are unlikley to warrant attention by river managers at the scale of this analysis.

For reach connections, 79.3% (1,392 km) and 18.3% (2,418 km) of Strategic and HRP reaches, respectively, had upstream and/or downstream connections with HRP and/or Conservation reaches (Tables 2 and S1). In comparison, 11.5% (669 km) of LRP reaches were upstream of HRP and/or Conservation reaches. Interestingly, all of the reach connections, apart from HRP between Conservation, are a small fraction, ranging from 0.1% to 0.6%, of total coastal NSW stream length. Within each category, apart from HRP between Conservation, there are less than 600 km of stream length identified for rehabilitation across coastal NSW.

For loci connections, 79.4% (1,394 km) of Strategic reaches were surrounded by HRP and/or Conservation reaches, or MRP and/or LRP reaches. In comparison, 53.6% (7,071 km) and 26.0% (8,476 km) of HRP and Conservation reaches, respectively, were surrounded by LRP and/or MRP reaches (Tables 2 and S1). A variety of connections were made with Strategic reaches, both upstream and downstream, and via their tributaries. Strategic reaches are connected to higher recovery potential Conservation and HRP reaches along 946 km of stream length. Strategic reaches are also connected to lower recovery potential MRP and LRP reaches along 935 km of stream length. Of these Strategic reaches, 486 km were connected to both higher and lower recovery potential reaches due to the broad range of recovery potential reaches in surrounding tributaries, and upstream and downstream connections. This resulted in the same Strategic reach being included in both ‘Strategic surrounded by HRP/Conservation’ and ‘Strategic surrounded by LRP/MRP’ categories. Where the same Strategic reach was included in more than one loci combination, the duplicate reach was manually subtracted from the total for ‘Strategic loci connections (presented in km and as % of total Strategic stream length)’, in order to remove the effects of this double counting (Table 2).

**Fig 4:**
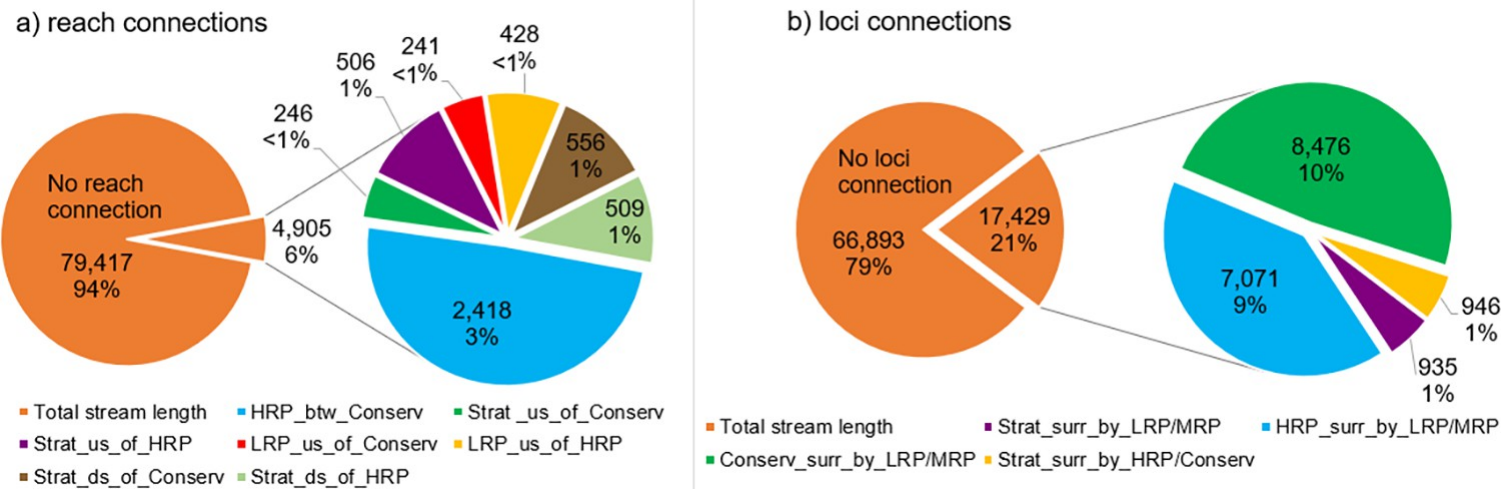
Reach and loci connections for NSW coastal catchments. (a) Reach connections and (b) loci connections, respectively, by connection type, showing total stream length per connection type in km, total coastal stream length without any connections in km, and % of total stream length.

**Fig 5:**
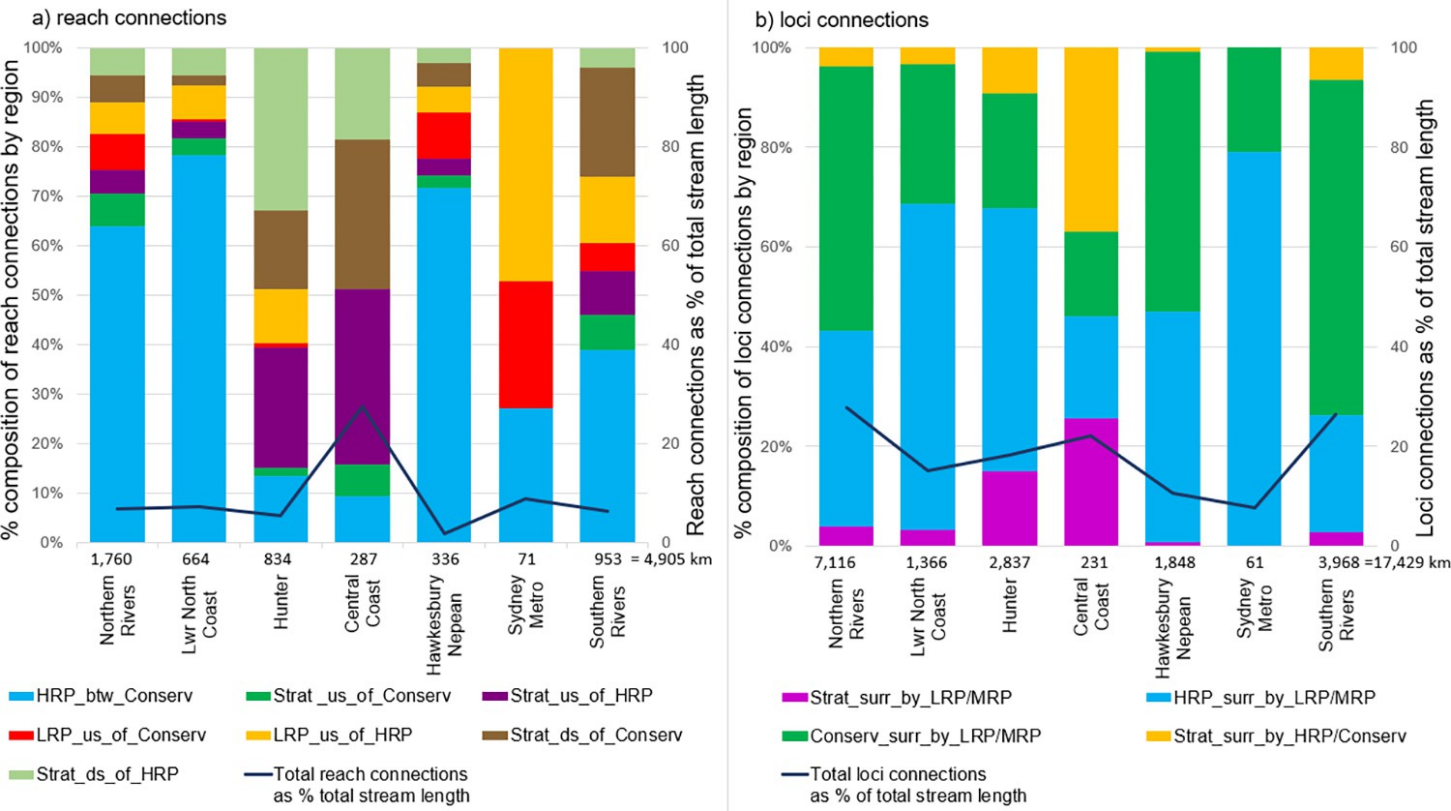
Reach and loci connections by region for NSW coastal catchments. (a) Reach connections and (b) loci connections, respectively, by region, showing % composition, % of total stream length, and total km of connections.

**Fig 6:**
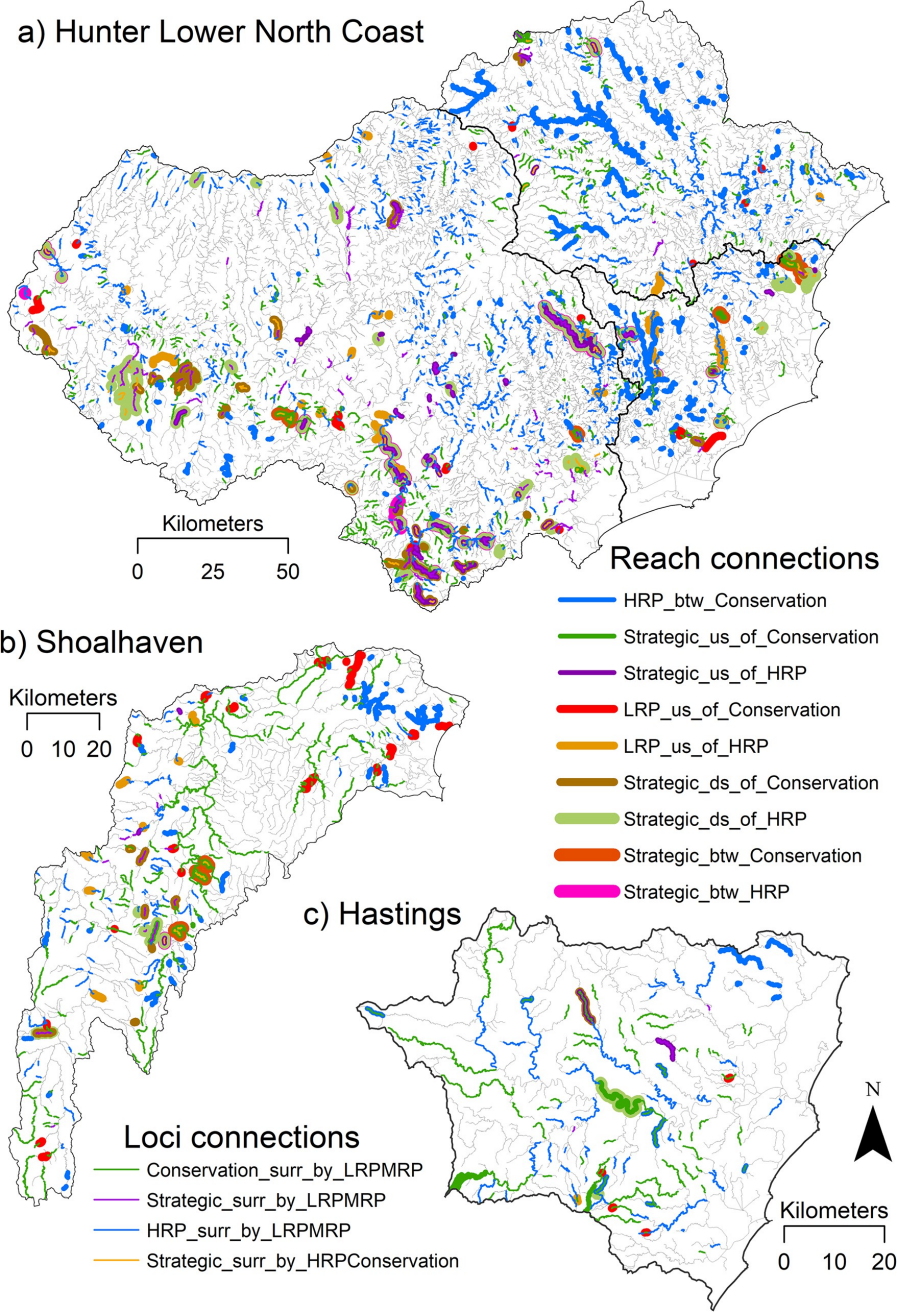
Reach and loci connections for (a) H-LNC (b) Shoalhaven and (c) Hastings catchments.

The spatial variability of reach connections varies across regions (Figs 3–6). Identified reach connections comprise only 6% of total coastal catchment stream length (Fig 4A). Of these, 49.3% have a HRP between Conservation pattern. When broken into regions, all except the Central Coast (27.6%) have less than 10% of their total stream length comprising identified reach connections (Fig 5). In some regions, such as Northern Rivers, Lower North Coast and Hawkesbury Nepean, these identified reach connections are dominated by HRP between Conservation, while other regions have a range of different types of reach connections (Fig 5). These small percentages highlight the benefits of this method to identify and extract reach connections from a large dataset, a task that would otherwise be laborious and overwhelming. The 94% of reach connections that are unallocated across coastal NSW do have a variety of possible connections, for example MRP upstream of LRP, but these have not been run as a permutation in this study. Loci connections are more prevalent across coastal NSW, comprising 21% of total stream length (Fig 4B). HRP surrounded by LRP and/or MRP, and Conservation surrounded by LRP and/or MRP, comprise 40.6% and 48.6% of the total loci connections, respectively, accounting for almost 90% of the loci connections (Fig 5). The percentage of loci connections across the regions varies from 7.7% to 27.9% of total stream length by region (Fig 5).

The results for the three representative catchments, Hastings, Hunter-Lower North Coast (H-LNC) and Shoalhaven, are shown in Fig 6 and Table 2. Hastings catchment is located in the Northern Rivers region and covers an area of 4,484 km^2^ and contains 3,624 km of stream length, has a predominantly agricultural land use and unregulated rivers. National Parks and State forests occur in the upper parts of the catchment [61–63]. H-LNC is located on the NSW mid north coast and covers an area of 34,400 km^2^, containing 24,551 km of stream length, with significant urban, mining, agricultural and forestry areas, and regulated rivers along the Hunter trunk stream [64]. Shoalhaven catchment is in the Southern Rivers region, covering 7,300 km^2^ with 4,518km of stream length, and is predominantly rural. The Shoalhaven River is partly regulated via the Tallowa Dam system [65].

A basic visual observation of these results shows that in some catchments there are a significant number of reach and loci connections to consider for conservation and rehabilitation, but in each place the type and extent of these connections varies. This distilled corridor analysis provides a much sharper and manageable set of results for each catchment. For example, in all coastal catchments of NSW (Table 2 and Fig 3B) there is 13,185 km of stream length assessed as HRP but only 2,418 km (or 18.0% of total HRP) creates an upstream and downstream reach connection with Conservation reaches. This is further reduced within the example catchments, to 633.6 km in H-LNC, 56.9 km in Hastings and 137.2 km in Shoalhaven (Table 2, Fig 6).

In H-LNC at least one of each type of reach or loci connection occurs. In this catchment, the connections are relatively long and cumulatively large. However, in Hastings and Shoalhaven catchments several types of connection do not occur and some connections are short and cumulatively small (<20 km of each type). As another example, the proportion of Conservation surrounded by LRP and/or MRP is higher in Shoalhaven compared to Hastings and H-LNC, 72.3%, 53.8% and 24.6% of total loci connections, respectively. This often represents Conservation upstream of LRP and/or MRP reaches.

## Discussion

### The need for landscape-scale river restoration and rehabilitation programs that work with fluvial corridors

The United Nations Sustainable Development Goals (SDGs) Life on Land and Clean Water and Sanitation have a 2020 target (now passed) to “ensure the conservation, restoration and sustainable use of terrestrial and inland freshwater ecosystems and their services” and a 2030 target to urgently “implement integrated water resources management at all levels”. To achieve these targets, the river management and restoration industry, globally, will need to work at much larger scales if the cumulative impacts of rehabilitation and restoration are to be manifest. Proactively maintaining or building corridors of river recovery, and working with the river to assist self-healing is one mechanism by which this can occur [11, 14]. However, such work cannot commence until corridors are identified and priorities set for their conservation or rehabilitation. Using a medical analogy, there is a need to identify ‘clogged or sick arteries’ in the body before undertaking surgery or a course of treatment to ‘unclog and reconnect’ them.

In many parts of the world, and for far too long, river restoration and rehabilitation has been carried out at the reach-scale and in an ad-hoc manner, treating reaches in isolation, and independent of their position in catchment [66, 67]. A shift to a more systemic approach to management of fluvial corridors and river systems, incorporates both local and catchment scale (or larger scale, region, State or National) rehabilitation decision-making, and is situated in the context of nature-based and process-based solutions [68]. Viewing the river environment as an interconnected ‘riverscape’, that adjusts, erodes, floods, functions, changes and evolves is imperative to this approach [69, 70]. Large scale ecosystem corridor conservation or building, of which fluvial corridors form a part, allows for the maintenance or creation of healthy functioning ecosystems [71, 72].

### How can corridor analysis be used to aid conservation and rehabilitation prioritisation and decision support systems?

Prioritisation frameworks or protocols, employed in numerous management settings, apply predetermined criteria to rank projects in a consistent manner. In natural resource management, criteria can cover a broad spectrum of competing environmental, economic and social factors, and encompass both quantitative and qualitative factors [73, 74]. At the more specific level, prioritisation protocols such as that used in the River Styles Framework (Fig 2A) have an environmental purpose, primarily focussed on improving geomorphic river condition by working in places where there is potential for recovery–either assisted or unassisted [7]. The method developed and used in this paper operationalises what is a largely a conceptual prioritisation protocol, and up-scales it from the reach-scale to the catchment and regional scale. It also leverages an open source database (of which there may be many similar examples elsewhere) and uses readily available GIS tools in a new way.

The corridor analysis undertaken in this study provides one example of how river managers can, early on in the evaluation process, assess, rank and prioritise projects based on environmental benefits and feasibility of conservation and/or rehabilitation. This method provides a way to identify where the ’easy win’ corridors might be, based on the prioritisation protocol used (Fig 1). The analysis provides a more focussed and manageable list of reaches and loci to work on. This can then be translated into decision-support systems to consider the cost and level of intervention required to achieve environmental outcomes and improvements in river condition. Identification of river reaches with enhanced potential for recovery enables river managers to build corridors of recovery and reduce fragmentation of the river system. In the framework developed here, prioritisation strategies based on environmental benefits and feasibility will differ depending on the particular reach or loci connection identified, its river type, its condition and the types of threatening processes or ‘problems’ that are evident [7, 18, 60].

For reach connections, prioritisation strategies could consider:

- *Working from upstream to downstream* where the benefits of rehabilitation will most positively impact downstream sections of rivers.
- *The total length of each recovery potential class relative to the total stream length in a catchment or region*. For example, there are 1755 km of Strategic reaches across coastal NSW catchments. This is <1.7% of the total NSW coastal stream length. This is relatively small length of Strategic stream length that could be considered a high priority for treatment across the entire region.
- *The number or length of fragmented reaches in each recovery potential connection*. For example, there are ~800 km and ~1015 km of Strategic-Conservation or Strategic-HRP connections across coastal NSW catchments that could be treated. This could be given a higher priority than say *a*. or *b*. depending on local decision-making and resource availability.
- *Whether a reach has been identified as a singular or multiple class connection*. For example, Strategic reaches that are multi-strategic (contain multiple colours on Fig 6) may be considered a higher priority for intervention than singularly classified Strategic reaches (contain single colours on Fig 6). It is likely that multi-strategic reaches have several ‘problems’ to address and/or multiple potential impacts on surrounding reaches. Left untreated the consequences could be detrimental. Conversely, treating these reaches could have multiple, cumulative positive feedback impacts on-site and off-site.

For loci connections, prioritisation strategies could consider:

- *The length of loci connection*. For example, where a HRP loci is surrounded by LRP and/or MRP reaches, the LRP/MRP reaches could be having a negative impact on the HRP loci. If the HRP loci is short and the LRP/MRP sections are long, then it is likely that the HRP loci will be negatively impacted and may deteriorate over time. However, if the HRP loci is long and the LRP/MRP sections are relatively short, then it may be desirable to intervene, building out from the HRP loci to trigger positive off-site impacts in the adjacent MRP/LRP reaches. Depending on local conditions, a threshold loci connection length could be used to focus the prioritisation further.
- *The recovery potential class of the loci connection*. For example, treating a Strategic loci containing a headcut would be considered a high priority to protect surrounding reaches with higher recovery potential. While this may involve treating a more degraded reach, the offsite-impacts of not intervening could be costly in the future.
- As for reach connections, *whether a loci connection is singularly or multi-classified*, with treatment of multi-classified loci potentially having multiple off-site benefits.

Additional considerations and nuances may also be required in these prioritisations to aid local decision-making. In the context of fluvial corridors, position in catchment will also be important. For example, a higher priority may be given to mid-catchment connections rather than connections in headwaters, or areas where there is a greater critical mass of upstream conservation or HRP reaches to work with. It could be decided that a short headwater Conservation-HRP-Conservation connection may have a lower cost:benefit than a long Conservation-HRP-Conservation connection in a mid-catchment location, or a critical mass of Conservation-HRP-Conservation connections in a number of subcatchments. The timing of interventions will also be important. For example, a decision could be made to postpone intervention in a LRP reach until positive off-site consequences of intervention elsewhere are manifest and impact on the recovery potential of that LRP reach [53].

Reach and loci connections can also be identified at a variety of scales. Rehabilitation of larger reach and loci connections are vital for regional and subnational landscape scale ecological connectivity [9, 10], and could be considered for rehabilitation by agencies with significant resources bases. There are also opportunities for smaller scale rehabilitation of shorter, more straightforward, connections which could be undertaken by individual landowners or community groups. In nature-based approaches to river management both strategies are needed.

While the corridor analysis conducted in this study focussed on the Conservation, Strategic and HRP classes and connections, the same process and workflow can be used to identify other connections. For example, river managers may wish to focus attention on MRP reaches once Conservation, Strategic and HRP reaches are treated. Alternatively, there may be other layers of information and broader considerations that need to be overlayed on the analysis to further differentiate and prioritise reaches. Importantly, the prioritised shortlists can be combined with local on-ground knowledge and management preferences, and considered in the context of other resource constraints and competing priorities, to ground decision making in a transparent and consistent manner [75].

Whilst it is important to identify and rank corridors of potential recovery, it is only the first step in achieving landscape scale rehabilitation outcomes, as the hard work of on-ground action needs to take place to conserve or build the corridors and create functionally health river systems. It is possible that by connecting corridors of river recovery, resilience can be built into river systems to mitigate against future floods and droughts, and declines in stream flow driven by anthropogenic disturbance or climate extremes [76]. Nature-based and process-based river rehabilitation tools are already available to make this a reality, the challenge now is to make it happen at-scale [14, 18].

## Conclusion

This study uses an Open Access database to systematically identify corridors of river recovery for conservation or rehabilitation in all coastal catchments of NSW, Australia. Reach and loci connections can be identified, at a variety of scales, from short and simple, to long and complex. These results can be used in decision support systems to prioritise river management activities and develop river management strategies at-scale. If agencies wish to work with recovery, the best rehabilitation benefits will accrue from building corridors of river recovery and working with reach and loci connections, that will build resilience into river systems and meet international obligations for water and river health.

## Supporting information

S1 Table Recovery potential, and reach and loci connections for each coastal region for NSW. The raw data in the Open Access NSW River Styles database has been processed to produce this summary.
(PDF)
