# Supplementary material for: Identifying corridors of river recovery in coastal NSW Australia, for use in river management decision support and prioritisation systems
Source: PLoS One. 2022 Jun 23;17(6):e0270285. doi: 10.1371/journal.pone.0270285 (PMC9223338; doi:10.1371/journal.pone.0270285)
Supplement: S1 Table — The raw data in the Open Access NSW River Styles database has been processed to produce this summary. (PDF) [file pone.0270285.s001.pdf]

S1 Table. Recovery potential, and reach and loci connections for each coastal region for NSW. The raw data in the Open Access NSW River Styles database has been processed to produce this summary.

Stream length [a] km	Northern Rivers	Lower North Coast	Hunter	Central Coast	Hawkesbury Nepean	Sydney Metro	Southern Rivers	Total stream length NSW coastal catchments
**Total freshwater stream length by recovery potential**	**25,549.7**	**9,081.0**	**15,467.3**	**1,044.3**	**17,365.5**	**787.1**	**15,047.6**	**84,342.6**
Conservation	11,169.0	4,375.1	2,940.1	385.4	5,133.6	361.4	8,203.2	32,567.6
Strategic	369.3	123.9	770.4	123.1	52.9	0.0	316.3	1,755.9
High recovery potential (HRP)	4,317.1	2,359.5	3,095.0	313.6	1,344.5	181.7	1,574.1	13,185.4
Moderate recovery potential (MRP)	8,275.8	1,845.2	5,831.6	75.0	2,359.8	63.8	3,666.0	22,117.0
Low recovery potential (LRP)	1,401.0	153.5	2,632.4	15.3	246.0	95.8	1,288.0	5,832.0
Null	17.5	224.0	197.9	132.0	8,228.9	84.4	0.0	8,884.7
**Total reach connections by region [a]**	**1,760.3**	**664.0**	**834.2**	**286.6**	**335.8**	**70.9**	**952.8**	**4,904.6**
HRP between Conservation	1,125.3	519.6	114.0	27.3	240.9	19.3	371.9	2,418.4
Strategic upstream of HRP	84.7	21.5	202.9	101.3	11.9	0.0	84.1	506.4
Strategic upstream of Conservation	115.7	23.8	12.2	18.0	8.2	0.0	68.0	246.0
LRP upstream of HRP	112.7	45.4	90.2	0.0	17.8	33.5	127.9	427.5
LRP upstream of Conservation	127.3	3.5	8.4	0.0	30.9	18.1	53.2	241.3
Strategic downstream of Conservation	96.4	12.9	133.4	87.0	15.9	0.0	210.4	556.1
Strategic downstream of HRP	98.2	37.3	273.1	52.9	10.2	0.0	37.3	508.9

**Additional reach connection types [a,b]**								
Strategic between HRP	27.7	11.3	118.6	43.4	0.8	0.0	3.9	205.6
Strategic between Conservation	2.0	5.0	8.5	4.9	5.5	0.0	46.9	72.9
**Total loci connections by region [a,b]**	**7,117.0**	**1,366.4**	**2,837.0**	**231.3**	**1,847.9**	**60.6**	**3,968.4**	**17,428.6**
Strategic surrounded by LRP and/or MRP	276.9	46.1	422.8	59.0	16.7	0.0	113.7	935.2
HRP surrounded by LRP and/or MRP	2,798.0	892.7	1,498.9	47.6	854.4	47.9	932.0	7,071.4
Conservation surrounded by LRP and/or MRP	3,769.2	380.3	654.5	39.1	960.0	12.7	2,660.6	8,476.5
Strategic surrounded by HRP and/or Conservation	272.8	47.3	260.8	85.6	16.9	0.0	262.1	945.6
**Additional loci connection types [a,b]**								
Strategic surrounded by HRP	181.6	32.1	201.9	61.9	10.8	0.0	106.8	595.2
Strategic surrounded by Conservation	201.5	29.4	127.0	55.3	9.3	0.0	237.7	660.2

[a] Only freshwater reaches included in the analysis. Tidal reaches excluded.

[b] Target reaches >1000 m
